# Solubility measurement and modeling of hydroxychloroquine sulfate (antimalarial medication) in supercritical carbon dioxide

**DOI:** 10.1038/s41598-023-34900-7

**Published:** 2023-05-19

**Authors:** Gholamhossein Sodeifian, Chandrasekhar Garlapati, Maryam Arbab Nooshabadi, Fariba Razmimanesh, Amirmuhammad Tabibzadeh

**Affiliations:** 1 Department of Chemical Engineering, Faculty of Engineering, University of Kashan, Kashan, 87317-53153 Iran; 2 Laboratory of Supercriritcal Fluids and Nanotechnology, University of Kashan, Kashan, 87317-53153 Iran; 3 Modeling and Simulation Centre, Faculty of Engineering, University of Kashan, Kashan, 87317-53153 Iran; 4 Department of Chemical Engineering, Puducherry Technological University, Puducherry, 605014 India; 5 Bolvar Ghotbe Ravandi, Kashan Branch, Islamic Azad University, Ostaadan Street, Kashan, 87159-98151 Iran

**Keywords:** Chemical engineering, Chemistry

## Abstract

A supercritical fluid, such as supercritical carbon dioxide (scCO_2_) is increasingly used for the micronization of pharmaceuticals in the recent past**.** The role of scCO_2_ as a green solvent in supercritical fluid (SCF) process is decided by the solubility information of the pharmaceutical compound in scCO_2_. The commonly used SCF processes are the rapid expansion of supercritical solution (RESS) and supercritical antisolvent precipitation (SAS). To implement micronization process, solubility of pharmaceuticals in scCO_2_ is required. Present study is aimed at both measuring and modeling of solubilities of hydroxychloroquine sulfate (HCQS) in scCO_2_. Experiments were conducted at various conditions (P = 12 to 27 MPa and T = 308 to 338 K), for the first time. The measured solubilities were found to be ranging between (0.0304 × 10^–4^ and 0.1459 × 10^–4^) at 308 K, (0.0627 × 10^–4^ and 0.3158 × 10^–4^) at 318 K, (0.0982 × 10^–4^ and 0.4351 × 10^–4^) at 328 K, (0.1398 × 10^–4^ and 0.5515 × 10^–4^) at 338 K. To expand the usage of the data, various models were tested. For the modelling task existing models (Chrastil, reformulated Chrastil, Méndez-Santiago and Teja (MST), Bartle et al., Reddy-Garlapati, Sodeifian et al., models) and new set of solvate complex models were considered. Among the all models investigated Reddy-Garlapati and new solvate complex models are able to fit the data with the least error. Finally, the total and solvation enthalpies of HCQS in scCO_2_ were calculated with the help of model constants obtained from Chrastil, reformulated Chrastil and Bartle et al., models.

## Introduction

There has been greater attention in the recent past about the application of supercritical carbon dioxide in micronization of pharmaceuticals^1–5^. The drug administration is decided by the size of the particle. As we know, intravenous drug delivery requires particles size ranging from 0.1 to 0.3 μm, inhalation delivery requires 1–5 μm and oral delivery requires 0.1–100 μm and the smaller the size of the particles, greater chance of a drug being absorbed by the human body, which helps in reducing the drug dosage^1^. Conventional particle reduction techniques result in products that are in the particulate range, for example jet mills provides product particles in the range 5–45 μm, hammer mill provides product particles in the range 25–600 μm, on the other hand supercritical fluids (SCFs) technology provides product particles in the range 0.1–600 μm^1^. But, to apply SCFs technology, solubility data of a specific drug in the desired SCF is required for the selection and design of suitable SCF process that reduces the particle size, thus the solubility will determine the operating condition of the process^6–8^. Present work is focused on the both solubility measurement and modelling of hydroxychloroquine sulfate (HCQS) in scCO_2_. This drug was originally developed in United States (US) to counter malaria in the year 1949^9,10^. HCQS is considered as better alternate to chloroquine, due to less toxicity. It is also used for the treatment of Rheumatoid Arthritis (RA) and Systemic Lupus Erythematosus (SLE)^9,11^ disease. A recent study conducted by Pishnamazi et al., on the solubility of chloroquinein scCO_2_ has inspired us to take up this task^12^. Since 1950, chloroquine has been in use to treat malaria, however, hydroxychloroquine, a chloroquine analogue has a better safety profile due to a hydroxyl group on the side chain and is used in the treatment of connective tissue disorders^13^. We believe this study helps to implement SCF technology to get the desired drug size particles of HCQS and which may help to reduce the drug dosage in treatment. To expand the use of solubility data, modelling is performed with literature models and new solvate complex models.

This work is carried out in two steps. In the first, HCQS solubility in scCO_2_ solvent is determined and in the second, data measured in the first stage are correlated with literature models. The models employed are solid–liquid equilibrium, Chrastil, reformulated Chrastil, Méndez-Santiago and Teja (MST), Bartle et al., Reddy-Garlapati, Sodeifian et al., models and three forms of new solvate complex models.

## Experimental

### Materials

HCQS was supplied by TEMAD Co., Active Pharmaceuticals in gradients, Mashhad, Iran (CAS Number: 747-36-4, mass purity > 99%). CO_2_ (CAS Number: 124-38-9, mass purity > 99.9%) was purchased from Fadak company, Kashan, Iran. All the relevant details are tabulated in Table 1.

**Table 1:** Molecular structure and physicochemical properties of used materials.

Compound	Formula	Structure	M_W_ (g/mol)	λ_max_ (nm)	CAS number	Minimum purity (%)
Hydroxychloroquine sulfate	C_18_H_28_ClN_3_O_5_S		434	220	747-36-4	99
Carbon dioxide	CO_2_		44.01		124-38-9	99.9

### Solubility measurement details

**Figure 1:**
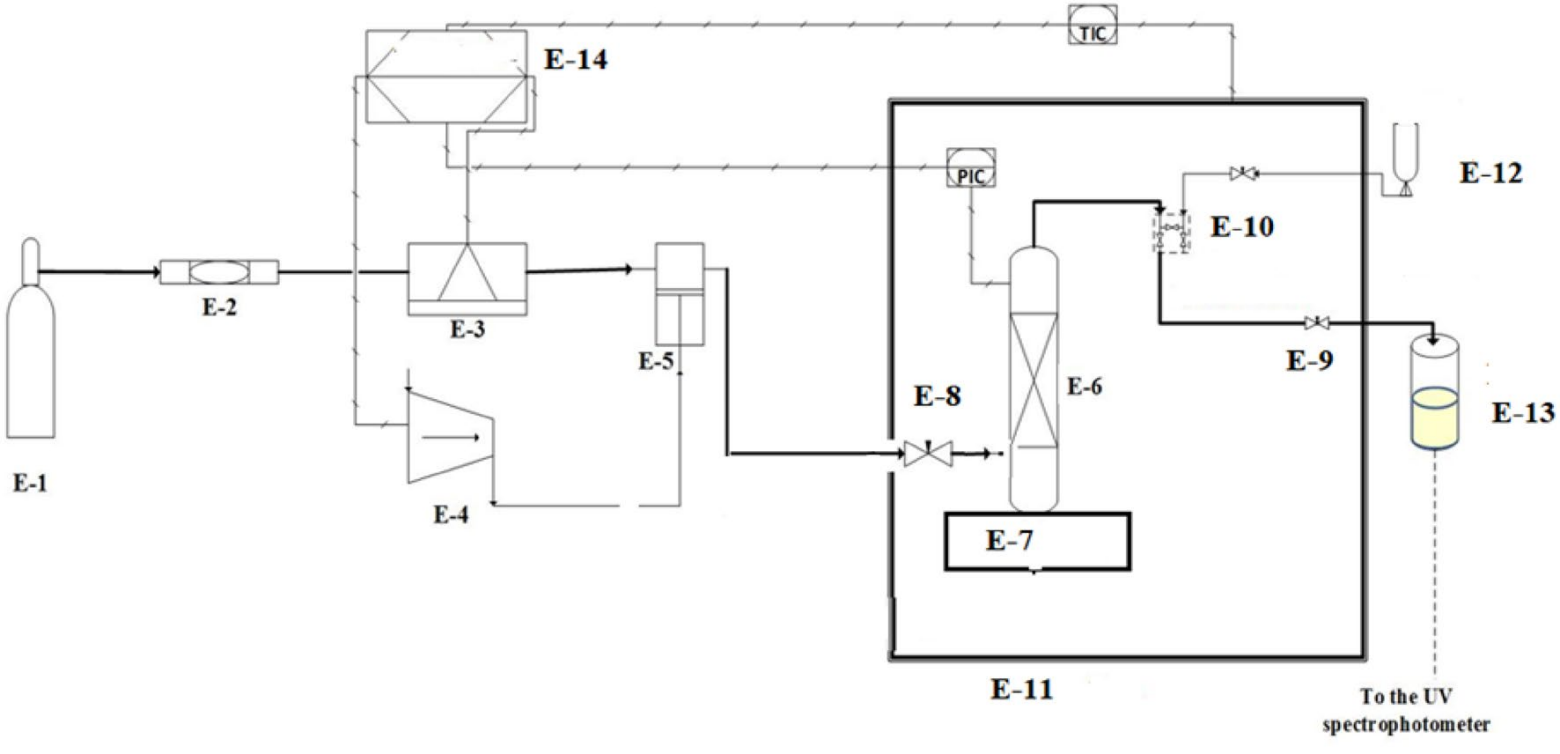
Experimental setup for solubility measurement, E1- CO_2_ cylinder; E2- Filter; E3- Refrigerator unit; E4- Air compressor; E5- High pressure pump; E6- Equilibrium cell; E7- Magnetic stirrer; E8- Needle valve; E9- Back-pressure valve; E10- Six-port, two position valve; E11- Oven; E12- Syringe; E13- Collection vial; and E14- Control panel.

SCF-solubility of the drugs was experimentally measured via two broad classes of saturated solution-based methods, where solubility measurement can be done either (1) statically or (2) dynamically^14^. In the present work, a UV–vis spectrophotometer was utilized to statically examine the equilibrium solubility data of HCQS in a setup presented in Fig. 1. This experimental setup has already been validated in our previous work with alpha-tocopherol and naphthalene^15^. The solubilities were measured with the help of an equilibrium cell. Thermodynamically, the method employed may be regarded as an isobaric-isothermal method^14^. All the measurements are taken by keeping system temperatures and pressures at the desired value with high precision (i.e., ± 0.1 K and ± 0.1 MPa). Complete details about the equipment and measurement procedures are presented elsewhere^15–35^. However, the description of the equipment and the methodology employed in establishing solubility data are briefly presented in this section. The scCO_2_ is pumped to the equilibrium cell and drug sample and scCO_2_ are thoroughly mixed and allowed to attain equilibrium. It is observed that the equilibrium is attained in 60 min. To ensure equilibrium solubility, the experiments are performed with fresh samples at various time intervals. For a specified temperature and pressure in each experiment, the drug sample is contacted with scCO_2_ and stirred thoroughly in an equilibrium cell until a specific time (5 min, 10 min, 20 min, 30 min, 40 min, 50 min and 60 min) and the solubility readings are recorded. It is observed that the solubility is independent of time after 30 min. However, for solubility measurement, the samples are collected after 1 h. For each sampling a 600 µL volume saturated sample is collected via a collection valve in a deionized water preloaded sample vial. After discharging of each sample, the sampling valve was cleaned with 1 ml of deionized water. Drug sample solubility is estimated with the following formula:

1 $$y_2 = \frac{n_{drug} }{n_{drug} + n_{CO_2 } }$$

where $y_{\scriptsize 2}$ is solubility of the drug in scCO_2_, ${n}_{\text{drug}}$ and ${n}_{{\text{CO}}_{2}}$ are number of moles of the drug and CO_2_ in the sampling loop, respectively.

The following formulas are used in data conversion

2 $$n_{{{\text{drug}}}} = \frac{{C_{s} \cdot V_{s} }}{{M_{s} }}$$

3 $$n_{{CO_{2} }} = \frac{{V_{1} \rho_{1} }}{{M_{{CO_{2} }} }}$$

where $C_{s}$ is concentration of the drug in g/L. V_1_ = 600 × 10^–6^ L and V_s_ = 5 × 10^–3^ L are sampling loop and vial volumes, respectively. $M_{s}$ and $M_{{CO_{2} }}$ are drug and CO_2_ molecular weights, respectively. ρ_1_ is density of scCO_2_ at each experimental condition, as presented in Table 2.

**Table 2:** Solubility of HCQC in scCO_2_ at various temperatures and pressures.

Temperature (K)^a^	Pressure (bar)^a^	Density of scCO_2_ (kg/m^3^)^56^	Mole fraction, y_2_ (×10^5^)	Experimental standard deviation, S(ȳ)×10^5^	Equilibrium solubility, S (g/L)	Expanded uncertainty of mole fraction, U (×10^5^)
308	120	769	0.0304	0.001	0.0231	0.0024
	150	817	0.0434	0.0014	0.0350	0.0034
	180	849	0.0592	0.0021	0.0497	0.0048
	210	875	0.1087	0.0041	0.0939	0.0095
	240	896	0.1231	0.0051	0.1088	0.0115
	270	914	0.1459	0.0061	0.1316	0.0138
318	120	661	0.0627	0.0011	0.0409	0.0037
	150	744	0.1098	0.0021	0.0806	0.0065
	180	791	0.1406	0.0041	0.1097	0.0104
	210	824	0.2029	0.0065	0.1650	0.0158
	240	851	0.2498	0.0121	0.2097	0.0264
	270	872	0.3158	0.0073	0.2718	0.0202
328	120	509	0.0982	0.0012	0.0494	0.0049
	150	656	0.1591	0.0051	0.1029	0.0125
	180	725	0.1970	0.0091	0.1409	0.0202
	210	769	0.2731	0.0019	0.2072	0.0127
	240	802	0.3154	0.0121	0.2496	0.0279
	270	829	0.4351	0.0183	0.3557	0.0413
338	120	388	0.1398	0.0018	0.0534	0.0074
	150	557	0.2297	0.0021	0.1263	0.0117
	180	652	0.2822	0.0115	0.1816	0.0262
	210	710	0.3299	0.0098	0.2312	0.0245
	240	751	0.3921	0.0041	0.2906	0.0192
	270	783	0.5515	0.0065	0.4262	0.0275

y_2_ and S are mole fraction of HCQS in scCO_2_ and equilibrium solubility (g/L), respectively. The experimental standard deviation was obtained by $$S\left( {y_{k} } \right) = \sqrt {\frac{{\mathop \sum \nolimits_{j = 1}^{n} \left( {y_{j} - \overline{y}} \right)^{2} }}{n - 1}}$$. Expanded uncertainty (U) and the relative combined standard uncertainty (u_combined_/y) are defined, respectively, as follows: (U) = k*u_*combined*_(k = 2) and $${\raise0.7ex\hbox{${u_{combined} }$} \!\mathord{\left/ {\vphantom {{u_{combined} } y}}\right.\kern-0pt} \!\lower0.7ex\hbox{$y$}} = \sqrt {\mathop \sum \limits_{i = 1}^{N} \left( {P_{i} u\left( {x_{i} } \right)/x_{i} } \right)^{2} }$$. In this research, *u(x*_*i*_*)* was considered as standard uncertainties of temperature, pressure, mole fraction, volumes and absorption. P_i_, sensitivity coefficients, are equal to the partial derivatives of y equation (Eq. 1) with respect to the *x*_*i*_.

^a^Standard uncertainty values are u(T) =  ± 0.1 K and u(p) =  ± 1 bar. The value of the coverage factor k = 2 was chosen on the basis of the level of confidence of approximately 95%.

Solubility is also described as

4 $$S = \frac{{C_{S} V_{s} }}{{V_{1} }}$$

The solubility and mole fraction relation is described as

5 $$S = \frac{{\rho M_{s}}}{{M_{{CO_{2}}}}}\frac{{y_{2}}}{{1 - y_{2}}}$$

The HCQS’s solubility is quantified in the UV–Visible spectrophotometer (Model UNICO-4802) with the help of deionized water $(conductivity \le 5\;\upmu {\text{S}}\;{\text{m}}^{ - 1} )$ as collection solvent with the wave length of 220 nm at UV spectrum.

## Modelling

Although there are several approaches in modelling solubility data, solid–gas equilibrium (known as equation of state (EoS) approach), solid–liquid equilibrium (SLE, also known as expanded liquid equilibrium approach) and empirical modelling are commonly used in literature for the data correlation^36–50^. For EoS approach critical properties of both solvent (scCO_2_) and solute (HCQS) are required, whereas the SLE approach requires only melting temperature and melting enthalpy of the solute, but empirical modelling doesn’t need any such information. HCQS is a typical compound and it is not possible to estimate its critical properties due to the presence of H_2_SO_4_ in its structure. Due to this fact EoS modelling is not persuaded. On the other hand, experimental melting temperature and melting enthalpies of HCQS are available; due to this reason expanded liquid modelling is explored. For empirical modelling the density of scCO_2_, and system temperature and pressure are required; since they are readily available, it is also persuaded here. For empirical modelling, six commonly used solubility models are considered and those models have a varying number of parameters in their equations ranging from three to seven. The modelling purpose of some empirical models is to check the self-consistency of the measured data and to estimation some of the thermodynamic information of the dissolution process. In general, solubility of solids in SCFs is visualized in terms of solvate complex formation; therefore, a new set of solvate complex models is proposed for the better data fitting/correlation. More details about all the models considered in this work are presented in the following subsections.

### Solid–liquid equilibrium (SLE) models

In this approach the HCQS solute is assumed to be infinitesimally dissolved in the scCO_2_ solvent. At equilibrium, the drug fugacity in solid phase is equal to that of fugacity of drug in scCO_2_ phase. From this criterion, the following solubility expression is proposed^51–57^.

6 $$y_{2} = \frac{1}{{\gamma_{2}^{\infty } }}\frac{{f_{2}^{S} }}{{f_{2}^{L} }}$$

From the literature, there are several models for this approach. However, Wilson activity coefficient model is considered for the data regression^58^.

We know from thermodynamics, $${\raise0.7ex\hbox{${f_{2}^{S} }$} \!\mathord{\left/ {\vphantom {{f_{2}^{S} } {f_{2}^{L} }}}\right.\kern-0pt} \!\lower0.7ex\hbox{${f_{2}^{L} }$}}$$ ratio is

7 $$\frac{{f_{2}^{S} }}{{f_{2}^{L} }} = \exp \left[ {\frac{{\Delta H_{2}^{m} }}{RT}\left( {\frac{T}{{T_{m} }} - 1} \right) - \int\limits_{{T_{m} }}^{T} {\frac{1}{{RT^{2} }}} \left[ {\int\limits_{{T_{m} }}^{T} {\left[ {\Delta C_{p} } \right]dT} } \right]dT} \right]$$

Simplified expression for $${\raise0.7ex\hbox{${f_{2}^{S} }$} \!\mathord{\left/ {\vphantom {{f_{2}^{S} } {f_{2}^{L} }}}\right.\kern-0pt} \!\lower0.7ex\hbox{${f_{2}^{L} }$}}$$ is^27^

8 $$\ln \left( {\frac{{f_{2}^{S} }}{{f_{2}^{L} }}} \right) = \frac{{\Delta H_{2}^{m} }}{RT}\left( {\frac{T}{{T_{m} }} - 1} \right)$$

The required $\gamma_{2}^{\infty }$ is obtained from Wilson model and the relevant expressions are as follows:

9 $$ln\left( {\gamma_{2}^{\infty } } \right) = 1 - \Lambda_{12} - ln\left( {\Lambda_{21} } \right)$$

where

10 $$\Lambda_{12} = \frac{{v_{2} }}{{v_{1} }}{\text{exp}}\left( { - \frac{{\lambda_{12} }}{RT}} \right)$$

11 $$\Lambda_{21} = \frac{{v_{1} }}{{v_{2} }}{\text{exp}}\left( { - \frac{{\lambda_{21} }}{RT}} \right)$$

12 $$\Lambda_{12} = v_{2} \rho_{c1} \rho_{r} exp\left( { - \frac{{\lambda_{12}^{^{\prime}} }}{{T_{r} }}} \right)$$

13 $$\Lambda_{12} = \frac{1}{{v_{2} \rho_{c1} \rho_{r} }}exp\left( { - \frac{{\lambda_{21}^{^{\prime}} }}{{T_{r} }}} \right)$$

$$\lambda_{12}^{^{\prime}} = \frac{{\lambda_{12} }}{{RT_{c1} }} \,{\text{and}}\,\lambda_{21}^{^{\prime}} = \frac{{\lambda_{21} }}{{RT_{c1} }}$$

14 $$lnv_{2} \left( {{\text{m}}^{3} \;{\text{mol}}^{ - 1} } \right) = \alpha_{2} ln\rho_{1} ({\text{kg}}\;{\text{m}}^{ - 3} ) + \beta_{2}$$

15 $$v_{2} = \alpha \rho_{r} + \beta$$

On combining Eqs. (10)–(15), we get

16 $$\Lambda_{12} = \left( {\alpha \rho_{r} + \beta } \right)\rho_{c1} \rho_{r} exp\left( { - \frac{{\lambda_{12}^{^{\prime}} }}{{T_{r} }}} \right)$$

17 $$\Lambda_{21} = \frac{1}{{ \left( {\alpha \rho_{r} + \beta } \right)\rho_{c1} \rho_{r} }}exp\left( { - \frac{{\lambda_{21}^{^{\prime}} }}{{T_{r} }}} \right)$$

$\lambda_{21}^{^{\prime}}$ and $\lambda_{12}^{^{\prime}}$ are the energies of interactions, where subscripts 1 and 2 denote solvent and solute, respectively.

Combining all the equations, finally, resulted in SLE model, i.e., Eq. (18)

18 $$y_{2} = {{\exp \left( {\frac{{\Delta H_{2}^{m} }}{RT}\left( {\frac{T}{{T_{m} }} - 1} \right)} \right)} \mathord{\left/ {\vphantom {{\exp \left( {\frac{{\Delta H_{2}^{m} }}{RT}\left( {\frac{T}{{T_{m} }} - 1} \right)} \right)} {\exp \left( {1 - \left( {\alpha \rho_{r} + \beta } \right)\rho_{c1} \rho_{r} \exp \left( { - \frac{{\lambda^{\prime}_{12} }}{{T_{r} }}} \right) - \ln \left( {\frac{1}{{\left( {\alpha \rho_{r} + \beta } \right)\rho_{c1} \rho_{r} }}\exp \left( { - \frac{{\lambda^{\prime}_{21} }}{{T_{r} }}} \right)} \right)} \right)}}} \right. \kern-0pt} {\exp \left( {1 - \left( {\alpha \rho_{r} + \beta } \right)\rho_{c1} \rho_{r} \exp \left( { - \frac{{\lambda^{\prime}_{12} }}{{T_{r} }}} \right) - \ln \left( {\frac{1}{{\left( {\alpha \rho_{r} + \beta } \right)\rho_{c1} \rho_{r} }}\exp \left( { - \frac{{\lambda^{\prime}_{21} }}{{T_{r} }}} \right)} \right)} \right)}}$$

### Commonly used empirical models

#### Chrastil's model^59^

In the year 1981, Josef Chrastil presented a model for the solubility of solids/liquids in SCF. The main basis of the model is a solvate complex formulation based on a simple reaction. According to Chrastil, solubility is explained by $c_{2} = c_{2} (\kappa ,\rho_{1} ,T)$, where $\kappa$ is association number, $\rho_{1}$ is solvent density (scCO_2_) and T is temperature. Chrastil proposed Eq. (19)

19 $$c_{2} = \rho_{1}^{\kappa } \exp \left( {A_{1} + \frac{{B_{1} }}{T}} \right)$$

An alternative from of Eq. (19) is Eq. (20)^60^

20 $$y_{2} = \frac{{\left( {\rho_{1} } \right)^{\kappa - 1} \exp \left( {A_{1} + \frac{{B_{1} }}{T}} \right)}}{{\left[ {1 + \left( {\rho_{1} } \right)^{\kappa - 1} \exp \left( {A_{1} + \frac{{B_{1} }}{T}} \right)} \right]}}$$

where y_2_ is solute solubility in mole fraction.

#### Reformulated Chrastil's model^61^

In the year 2009, Garlpati and Madras has reformulated Chrastil's model and it is expressed by Eq. (21), where solubility is explained by $y_{2} = y_{2} \left( {\kappa^{\prime},\rho_{1} ,T} \right)$, in which $\kappa^{\prime}$ is association number, $\rho_{1}$ is solvent density (scCO_2_) and T is temperature.

21 $$y_{2} = \left( {\frac{{RT\rho_{1} }}{{M_{ScF} f^{ \bullet } }}} \right)^{{\kappa^{\prime} - 1}} \exp \left( {A_{2} + \frac{{B_{2} }}{T}} \right)$$

in Eq. (21), R denotes universal constant of ideal gas, $M_{ScF}$ is molecular weight of solvent, $f^{ * }$ is reference pressure and $A_{2} \;$ and $\;B_{2}$ are the Reformulated model constants.

#### Méndez-Santiago and Teja (MST) model^62^

The MST model is used to check the consistency of the data. All the data falls around a single straight line when $T\ln \left( {y_{2} P} \right) - C_{3} T$ versus $\rho_{1}$ is established. MST model is expressed as Eq. (22)

22 $$T\ln \left( {y_{2} P} \right) = A_{3} + B_{3} \rho_{1} + C_{3} T$$

where *A*_*3*_ to *C*_*3*_ are the model constants.

#### Bartle et al., model^63^

The solute sublimation enthalpy is calculated with Bartle et al., model and it is stated as

23 $$\ln \left( {\frac{{y_{2} P}}{{P_{ref} }}} \right) = A_{4} + \frac{{B_{4} }}{T} + C_{4} \left( {\rho_{1} - \rho_{ref} } \right)$$

where *A*_*4*_ to *C*_*4*_ are the model constants. Using the constant $B_{4}$, sublimation enthalpy is calculated (i.e., $\Delta_{sub} H = - B_{4} R$).

#### Reddy-Garlapati model^64^

It is the model developed based on the degree of freedom analysis based on drug compounds. According to this model, solubility, $y_{2} = y_{2} \left( {T_{r} ,P_{r} } \right)$, is expressed as Eq. (24), where $T_{r}$ and $P_{r}$ are reduced temperature and pressures, respectively.

24 $$y_{2} = \left( {A_{5} + B_{5} P_{r} + C_{5} P_{r}^{2} } \right)T_{r} + (D_{5} + E_{5} P_{r} + F_{5} P_{r}^{2} )$$

where $A_{5}$ to $F_{5}$ are the model constants.

#### Sodeifian et al., model^65^

It is another recently proposed empirical model. According to this model, solubility,$y_{2} = y_{2} \left( {T,P,\rho_{1} } \right)$, can be calculated by

25 $$y_{2} = A_{6} + B_{6} \frac{{P^{2} }}{T} + C_{6} \ln \left( {\rho_{1} T} \right) + D_{6} \rho_{1} \ln \left( {\rho_{1} } \right) + E_{6} P\ln \left( T \right) + F_{6} \frac{{\ln \left( {\rho_{1} } \right)}}{T}$$

where $A_{6}$ to $F_{6}$ are the model constants.

### New solvate complex models^66^

The solubility is visualized via solvate complex formation. The following simple reversible reaction is considered for the formation of solvate complex $AB_{\kappa }$, in which ‘*A*’ is designated as solute and the letter ‘*B*’ is designated as solvent in SCF mixture.

26 $$A + \kappa^{\prime\prime}B \Leftrightarrow AB_{\kappa }$$

At equilibrium

27 $$K_{f} = \frac{{\left( {{{\hat{f}_{{AB_{\kappa } }} } \mathord{\left/ {\vphantom {{\hat{f}_{{AB_{\kappa } }} } {f_{{AB_{\kappa } }}^{ * } }}} \right. \kern-0pt} {f_{{AB_{\kappa } }}^{ * } }}} \right)_{SP} }}{{\left( {{{\hat{f}_{A} } \mathord{\left/ {\vphantom {{\hat{f}_{A} } {f_{A}^{ * } }}} \right. \kern-0pt} {f_{A}^{ * } }}} \right)_{Solid} \;\left( {{{\hat{f}_{B} } \mathord{\left/ {\vphantom {{\hat{f}_{B} } {f_{B}^{ * } }}} \right. \kern-0pt} {f_{B}^{ * } }}} \right)^{\kappa }_{SP} }}$$

where the subscript ‘Solid’ is designated as solid phase and subscript ‘SP’ is designated as solvent phase and superscript ‘$*$’ is reference state.

For each species the fugacity’s expressions are

28 $$\hat{f}_{A} = y_{A} \hat{\phi }_{A} P$$

29 $$\hat{f}_{B} = y_{B} \hat{\phi }_{B} P$$

30 $$\hat{f}_{{AB_{\kappa } }} = y_{{AB_{\kappa } }} \hat{\phi }_{{AB_{\kappa } }} P$$

31 $$f_{{AB_{\kappa } }}^{ * } = \phi_{{AB_{\kappa } }}^{ * } \;P^{ * }$$

32 $$f_{A}^{ * } = \phi_{A}^{ * } \;P^{ * }$$

33 $$f_{B}^{ * } = \phi_{B}^{ * } \;P^{ * }$$

At equilibrium in vapour phase only two things exit: one is solvent and the other is solvate complex thus

34 $$y_{B} + y_{ {AB_{\kappa } }} = 1$$

where $y_{B} , y_{{AB_{\kappa } }}$ are respective mole fractions.

If we assume the standard state of species ‘*A’* is pure under same system conditions. Then

35 $$\hat{f}_{A} = f_{A}$$

Fugacity of species ‘*A*’ can be written as

36 $$f_{A} = P_{A}^{Sub} \exp \left[ {\frac{{v_{A} \left( {P - P_{A}^{Sub} } \right)}}{RT}} \right]$$

From thermodynamics, equilibrium constant is function of salvation enthalpy as Eq. (37)

37 $$\ln \left( {K_{f} } \right) = {{\Delta H_{S} } \mathord{\left/ {\vphantom {{\Delta H_{S} } {RT + q_{s} }}} \right. \kern-0pt} {RT + q_{s} }}$$

Sublimation pressure can be expressed as Eq. (38)

38 $$\ln \left( {P_{A}^{Sub} } \right) = \alpha^{\prime} - {{\beta^{\prime}} \mathord{\left/ {\vphantom {{\beta^{\prime}} T}} \right. \kern-0pt} T}$$

Substituting Eqs. (35)–(38) into Eq. (27) along with two simplifications gives Eq. (39)

One assumption that ${{v_{A} P} \mathord{\left/ {\vphantom {{v_{A} P} {RT}}} \right. \kern-0pt} {RT}}$ is expressed as ${{Zv_{A} \rho_{1} } \mathord{\left/ {\vphantom {{Zv_{A} \rho_{1} } M}} \right. \kern-0pt} M}$ where $\rho_{1}$ is the density of the supercritical phase and the second assumption is refereed to negligible term ${{v_{A} P_{A}^{Sub} } \mathord{\left/ {\vphantom {{v_{A} P_{A}^{Sub} } {RT}}} \right. \kern-0pt} {RT}}$(~ 10^–8^–10^–9^) [since the sublimation pressures are very low and their order is about (~ 10^–3^–10^–4^) and the drug molar volume is also about the order (~ 10^–4^)]^67–70^.

39 $$\ln \left( {y_{{AB_{\kappa } }} } \right) - \kappa \ln \left( {y_{B} } \right) + \left( {1 - \kappa^{\prime\prime}} \right)\ln \left( {{P \mathord{\left/ {\vphantom {P {P^{ * } }}} \right. \kern-0pt} {P^{ * } }}} \right) = {L \mathord{\left/ {\vphantom {L T}} \right. \kern-0pt} T} + M\rho + N$$

where $$L = \left( {{{\Delta H_{s} } \mathord{\left/ {\vphantom {{\Delta H_{s} } R}} \right. \kern-0pt} R} - \beta^{\prime}} \right)$$, $$M = \left( { - {{ZV_{A} } \mathord{\left/ {\vphantom {{ZV_{A} } M}} \right. \kern-0pt} M}} \right)$$ and $$N = \ln \left( {\frac{{\left( {\varphi_{A}^{ * } } \right)\left( {{{\hat{\varphi }_{{AB_{\kappa } }} } \mathord{\left/ {\vphantom {{\hat{\varphi }_{{AB_{\kappa } }} } {\varphi_{{AB_{\kappa } }}^{ * } }}} \right. \kern-0pt} {\varphi_{{AB_{\kappa } }}^{ * } }}} \right)}}{{\left( {{{\hat{\varphi }_{B} } \mathord{\left/ {\vphantom {{\hat{\varphi }_{B} } {\varphi_{B}^{ * } }}} \right. \kern-0pt} {\varphi_{B}^{ * } }}} \right)^{\kappa } }}} \right) + \ln P^{ * } + q_{s} - \alpha^{\prime}$$

Equation (39) is rearranged as Eq. (40)

40 $$y_{{AB_{\kappa } }} = \left( {y_{B} } \right)^{{\kappa^{\prime\prime}}} \left( {{P \mathord{\left/ {\vphantom {P {P^{ * } }}} \right. \kern-0pt} {P^{ * } }}} \right)^{{\left( {\rlap{--} \kappa^{\prime\prime} - 1} \right)}} \exp \left( {{L \mathord{\left/ {\vphantom {L T}} \right. \kern-0pt} T} + M\rho + N} \right)$$

Further, on rearranging Eq. (40), we get Eq. (41)

41 $${{y_{{AB_{\kappa } }} } \mathord{\left/ {\vphantom {{y_{{AB_{\kappa } }} } {\left( {1 - y_{{AB_{\kappa } }} } \right)^{{\kappa^{\prime\prime}}} }}} \right. \kern-0pt} {\left( {1 - y_{{AB_{\kappa } }} } \right)^{{\kappa^{\prime\prime}}} }} = \left( {{P \mathord{\left/ {\vphantom {P {P^{ * } }}} \right. \kern-0pt} {P^{ * } }}} \right)^{{\left( {\kappa^{\prime\prime} - 1} \right)}} \exp \left( {{L \mathord{\left/ {\vphantom {L T}} \right. \kern-0pt} T} + M\rho + N} \right)$$

Applying binomial expansion to the left side term gives Eq. (41)

42 $${{y_{{AB_{\kappa } }} } \mathord{\left/ {\vphantom {{y_{{AB_{\kappa } }} } {\left( {1 - \kappa^{\prime\prime}\;y_{{AB_{\kappa } }} } \right)}}} \right. \kern-0pt} {\left( {1 - \kappa^{\prime\prime}\;y_{{AB_{\kappa } }} } \right)}} = \left( {{P \mathord{\left/ {\vphantom {P {P^{ * } }}} \right. \kern-0pt} {P^{ * } }}} \right)^{{\left( {\kappa^{\prime\prime\prime} - 1} \right)}} \exp \left( {{L \mathord{\left/ {\vphantom {L T}} \right. \kern-0pt} T} + M\rho + N} \right)$$

We know solubility, $y_{2}$, is related to cluster mole fraction $y_{{AB_{\kappa } }}$ as Eq. (43)^68,69^

43 $$y_{2} = {{y_{{AB_{\kappa } }} } \mathord{\left/ {\vphantom {{y_{{AB_{\kappa } }} } {(1 + \kappa^{\prime\prime}\;y_{{AB_{\kappa } }} )}}} \right. \kern-0pt} {(1 + \kappa^{\prime\prime}\;y_{{AB_{\kappa } }} )}}$$

Thus the solubility is expressed as Eq. (44)

44 $$y_{2} = {{\left( {{P \mathord{\left/ {\vphantom {P {P^{ * } }}} \right. \kern-0pt} {P^{ * } }}} \right)^{{(\kappa^{\prime\prime} - 1)}} \exp \left( {{L \mathord{\left/ {\vphantom {L T}} \right. \kern-0pt} T} + M\rho + N} \right)} \mathord{\left/ {\vphantom {{\left( {{P \mathord{\left/ {\vphantom {P {P^{ * } }}} \right. \kern-0pt} {P^{ * } }}} \right)^{{(\kappa^{\prime\prime} - 1)}} \exp \left( {{L \mathord{\left/ {\vphantom {L T}} \right. \kern-0pt} T} + M\rho + N} \right)} {\left[ {1 + \kappa^{\prime\prime}\left[ {\left( {{P \mathord{\left/ {\vphantom {P {P^{ * } }}} \right. \kern-0pt} {P^{ * } }}} \right)^{{(\kappa^{\prime\prime} - 1)}} \exp \left( {{L \mathord{\left/ {\vphantom {L T}} \right. \kern-0pt} T} + M\rho + N} \right)} \right]} \right]}}} \right. \kern-0pt} {\left[ {1 + \kappa^{\prime\prime}\left[ {\left( {{P \mathord{\left/ {\vphantom {P {P^{ * } }}} \right. \kern-0pt} {P^{ * } }}} \right)^{{(\kappa^{\prime\prime} - 1)}} \exp \left( {{L \mathord{\left/ {\vphantom {L T}} \right. \kern-0pt} T} + M\rho + N} \right)} \right]} \right]}}$$

$\kappa^{\prime\prime}$ is a function of reduced density, given by Eq. (45)^71^

45 $$\kappa^{\prime\prime} = a_{1} + a_{2} \rho_{r} + a_{3} \rho_{r}^{2}$$

Combining Eqs. (44) and (45) results in Eq. (46)

46 $$y_{2} = {{\left( {{P \mathord{\left/ {\vphantom {P {P^{ * } }}} \right. \kern-0pt} {P^{ * } }}} \right)^{{(a_{1} + a_{2} \rho_{r} + a_{3} \rho_{r}^{2} - 1)}} \exp \left( {{L \mathord{\left/ {\vphantom {L T}} \right. \kern-0pt} T} + M\rho + N} \right)} \mathord{\left/ {\vphantom {{\left( {{P \mathord{\left/ {\vphantom {P {P^{ * } }}} \right. \kern-0pt} {P^{ * } }}} \right)^{{(a_{1} + a_{2} \rho_{r} + a_{3} \rho_{r}^{2} - 1)}} \exp \left( {{L \mathord{\left/ {\vphantom {L T}} \right. \kern-0pt} T} + M\rho + N} \right)} {\left[ {1 + \left( {a_{1} + a_{2} \rho_{r} + a_{3} \rho_{r}^{2} } \right)\left[ {\left( {{P \mathord{\left/ {\vphantom {P {P^{ * } }}} \right. \kern-0pt} {P^{ * } }}} \right)^{{(a_{1} + a_{2} \rho_{r} + a_{3} \rho_{r}^{2} - 1)}} \exp \left( {{L \mathord{\left/ {\vphantom {L T}} \right. \kern-0pt} T} + M\rho + N} \right)} \right]} \right]}}} \right. \kern-0pt} {\left[ {1 + \left( {a_{1} + a_{2} \rho_{r} + a_{3} \rho_{r}^{2} } \right)\left[ {\left( {{P \mathord{\left/ {\vphantom {P {P^{ * } }}} \right. \kern-0pt} {P^{ * } }}} \right)^{{(a_{1} + a_{2} \rho_{r} + a_{3} \rho_{r}^{2} - 1)}} \exp \left( {{L \mathord{\left/ {\vphantom {L T}} \right. \kern-0pt} T} + M\rho + N} \right)} \right]} \right]}}$$

To reduce the number constants in Eq. (46), we have a choice to choose $\kappa$ as a linear function of reduced density or as constant. Thus, we get the following two reduced solubility expressions.

47 $$y_{2} = {{\left( {{P \mathord{\left/ {\vphantom {P {P^{ * } }}} \right. \kern-0pt} {P^{ * } }}} \right)^{{(a_{1} + a_{2} \rho_{r} - 1)}} \exp \left( {{L \mathord{\left/ {\vphantom {L T}} \right. \kern-0pt} T} + M\rho + N} \right)} \mathord{\left/ {\vphantom {{\left( {{P \mathord{\left/ {\vphantom {P {P^{ * } }}} \right. \kern-0pt} {P^{ * } }}} \right)^{{(a_{1} + a_{2} \rho_{r} - 1)}} \exp \left( {{L \mathord{\left/ {\vphantom {L T}} \right. \kern-0pt} T} + M\rho + N} \right)} {\left[ {1 + \left( {a_{1} + a_{2} \rho_{r} } \right)\left[ {\left( {{P \mathord{\left/ {\vphantom {P {P^{ * } }}} \right. \kern-0pt} {P^{ * } }}} \right)^{{(a_{1} + a_{2} \rho_{r} - 1)}} \exp \left( {{L \mathord{\left/ {\vphantom {L T}} \right. \kern-0pt} T} + M\rho + N} \right)} \right]} \right]}}} \right. \kern-0pt} {\left[ {1 + \left( {a_{1} + a_{2} \rho_{r} } \right)\left[ {\left( {{P \mathord{\left/ {\vphantom {P {P^{ * } }}} \right. \kern-0pt} {P^{ * } }}} \right)^{{(a_{1} + a_{2} \rho_{r} - 1)}} \exp \left( {{L \mathord{\left/ {\vphantom {L T}} \right. \kern-0pt} T} + M\rho + N} \right)} \right]} \right]}}$$

and

48 $$y_{2} = {{\left( {{P \mathord{\left/ {\vphantom {P {P^{ * } }}} \right. \kern-0pt} {P^{ * } }}} \right)^{{(\kappa^{\prime\prime} - 1)}} \exp \left( {{L \mathord{\left/ {\vphantom {L T}} \right. \kern-0pt} T} + M\rho + N} \right)} \mathord{\left/ {\vphantom {{\left( {{P \mathord{\left/ {\vphantom {P {P^{ * } }}} \right. \kern-0pt} {P^{ * } }}} \right)^{{(\kappa^{\prime\prime} - 1)}} \exp \left( {{L \mathord{\left/ {\vphantom {L T}} \right. \kern-0pt} T} + M\rho + N} \right)} {\left[ {1 + \left( {\kappa^{\prime\prime}} \right)\left[ {\left( {{P \mathord{\left/ {\vphantom {P {P^{ * } }}} \right. \kern-0pt} {P^{ * } }}} \right)^{{(\kappa^{\prime\prime} - 1)}} \exp \left( {{L \mathord{\left/ {\vphantom {L T}} \right. \kern-0pt} T} + M\rho + N} \right)} \right]} \right]}}} \right. \kern-0pt} {\left[ {1 + \left( {\kappa^{\prime\prime}} \right)\left[ {\left( {{P \mathord{\left/ {\vphantom {P {P^{ * } }}} \right. \kern-0pt} {P^{ * } }}} \right)^{{(\kappa^{\prime\prime} - 1)}} \exp \left( {{L \mathord{\left/ {\vphantom {L T}} \right. \kern-0pt} T} + M\rho + N} \right)} \right]} \right]}}$$

In literature, related solubility expression is proposed by Rajasekhar and Madras^67^ as

49 $$y_{2} = \left( {\frac{P}{{P^{ * } }}} \right)^{\begin{subarray}{l} \left( {\kappa^{\prime\prime\prime} - 1} \right) \\ \end{subarray} } \exp \left( {{{L^{\prime}} \mathord{\left/ {\vphantom {{L^{\prime}} {T + M^{\prime}\rho + N^{\prime}}}} \right. \kern-0pt} {T + M^{\prime}\rho + N^{\prime}}}} \right)$$

The correlating abilities and evaluation of the new models (Eqs. 46–48) and existing model (Eq. 49) are also carried out in this work.

The data fitting to the models are performed by an objective function Eq. (50)^72^

50 $$OF = \sum\limits_{i = 1}^{N} {\frac{{\left| {y_{2i}^{\exp } - y_{2i}^{calc} } \right|}}{{y_{2i}^{\exp } }}}$$

The obtained deviations are indicated in terms of an average absolute relative deviation percentage (AARD%).

51 $${\text{AARD}}\% = \left( {{\raise0.7ex\hbox{${100}$} \!\mathord{\left/ {\vphantom {{100} {N_{i} }}}\right.\kern-0pt} \!\lower0.7ex\hbox{${N_{i} }$}}} \right)\sum\limits_{i = 1}^{N} {\frac{{\left| {y_{2i}^{\exp } - y_{2i}^{cal} } \right|}}{{y_{2i}^{\exp } }}}$$

The entire regression task was done using (MATLAB 2019a®) version software, also this can be performed by nonlinear regression methods with the same results^84,85^.

## Results and discussion

**Figure 2:**
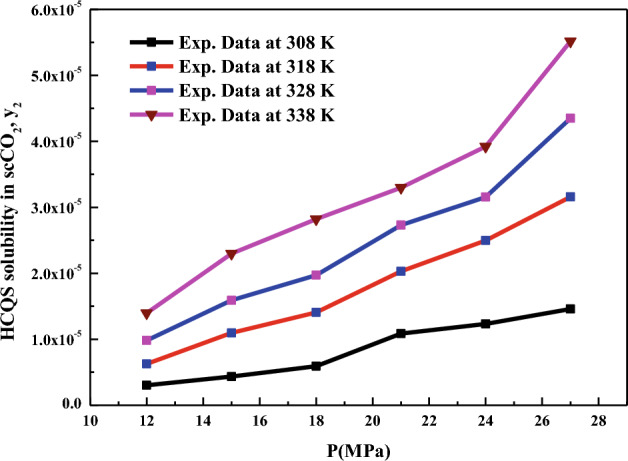
Solubility isotherms of HCQS in scCO_2_.

The experimental device used for the measurement of HCQS solubility in scCO_2_ is accurate and reliable. It was successfully tested to reproduce the solubilities of naphthalene in scCO_2_ and alpha-tocopherol in scCO_2_ systems and the same was reported in our earlier work^15^. The solubility values reported for alpha-tocopherol in our previous work, are the average of three replicate measurements with relative standard deviations lower than 5.7%^15^. The solubility values of naphthalene reported in our previous work are also the average of three replicate measurements with relative standard deviations lower than 6.5%^15^. Table 2 shows the measured solubilities of HCQS in scCO_2_ at various conditions and the density of the scCO_2_, obtained from the NIST data base^73^. From solubility data, it is clear that present system (HCQS+scCO_2_), does not exhibit the usual retrograde phenomenon. Affecting solvent density and solute power, the temperature was found to impose a dual impact on solubility in scCO_2_ depending on how the solute vapor pressure and solvent density are balanced. In this respect, increasing the solution temperature may enhance the solute vapor pressure, thereby contributing to stronger solvating power of SCF. At the same time, a rise of temperature may lower the scCO_2_ density which is known to depreciate the overall solvating power of the fluid. The mole fraction versus pressure isotherms on Fig. 2 suggest an enhancement in the solubility of drug upon elevating the temperature. This proves the dominant role of the solute vapor pressure in determining the solubility behavior irrespective of the pressure. Reports by other researchers confirm the results of the present work regarding the effect of temperature on the solubility in scCO_2_^74–76^. From Table 2 and Fig. 2, it is clear that when temperature is raised from 308 to 338 K, there is a clear indication of a rise in solubility from 0.0304 × 10^−4^ to 0.1398 × 10^−4^ at 12 MPa (i.e., 4.6 folds’ increase) and at 27 MPa from 0.1459 × 10^−4^ to 0.5515 × 10^−4^ (i.e., 3.8 folds increase). At the same time, at 12 MPa, the density of scCO_2_ changes from 769 to 338 kg m^−3^ at 308 and 338 K, respectively. Similarly, the density of scCO_2_ at 27 MPa changes from 914 to 783 kg m^−3^ at 308 and 338 K, respectively; which means that there is decrease in density at 12 MPa (low pressure) (i.e., 338/769 = 0.4395) and there is increase in density to some extent at 27 MPa (higher pressure) (i.e., 783/914 = 0.8567). Thus, the solubility behavior of HCQS in scCO_2_ is highly nonlinear. This kind of high nonlinearity behavior has been observed with amlodipine besylate-scCO_2_ in the recent past^28^. This kind of high nonlinearity behavior can’t be captured with simple models. Thus models having more adjustable constants are required to fit the data and this would augment the justification for the need of development of new models.

**Figure 3:**
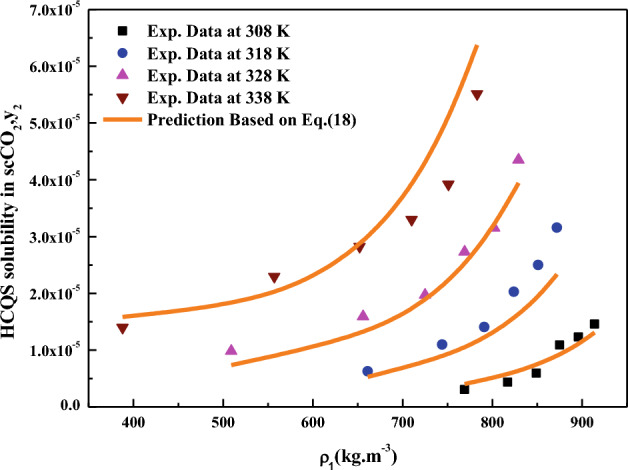
HCQS solubility in scCO_2_ versus ρ_1_. Symbols are experimental data points. Solid lines are calculated from SLE model (Eq. 18).

**Figure 4:**
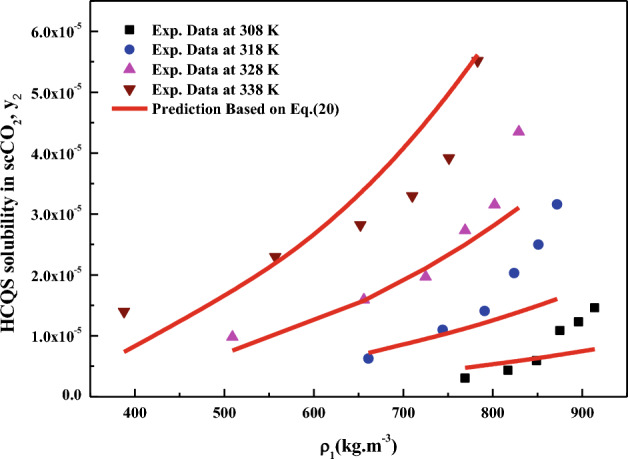
HCQS solubility in scCO_2_ versus ρ_1_. Symbols are experimental data points. Solid lines are calculated from Chrastil's model (Eq. 20).

**Figure 5:**
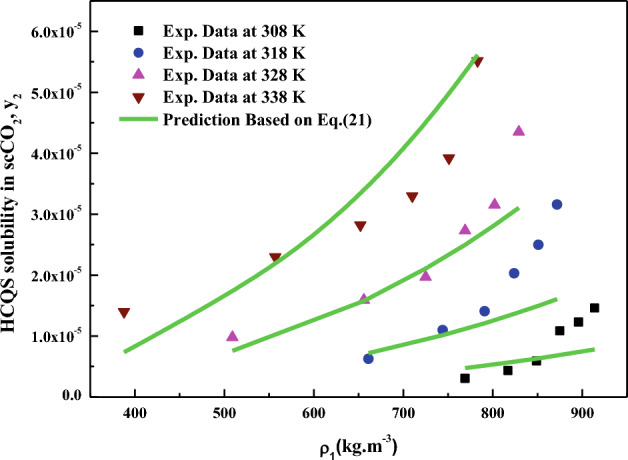
HCQS solubility in scCO_2_ versus ρ_1_. Symbols are experimental data points. Solid lines are calculated from SLE model (Eq. 21).

**Figure 6:**
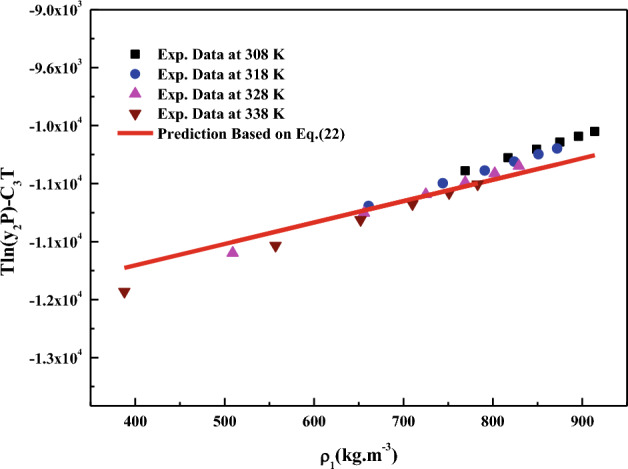
Self-consistency plot based on MST model (Eq. 22).

**Figure 7:**
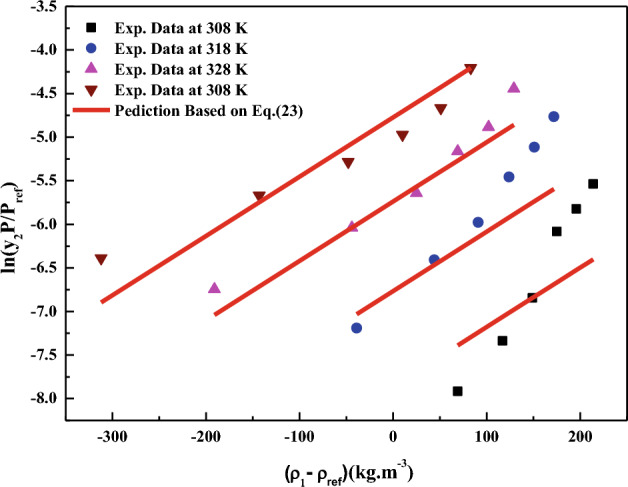
ln(y_2_ P/P_ref_) versus (ρ_1_ − ρ_ref_). Symbols are experimental data points. Solid lines are calculated from Bartle et al., model (Eq. 23).

**Figure 8:**
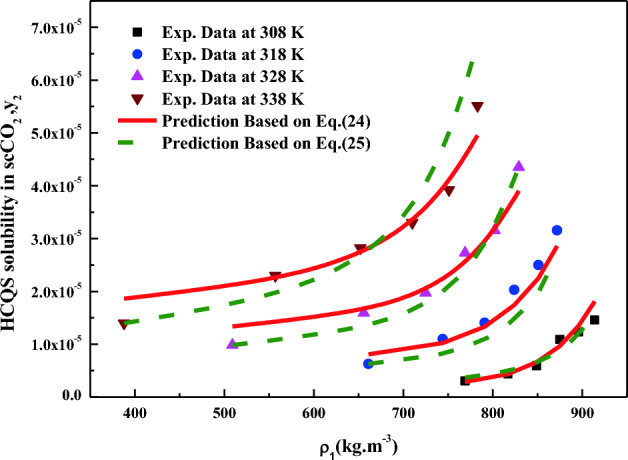
HCQS solubility in scCO_2_ versus ρ_1_.Symbols are experimental data points. Solid lines and broken linesare calculated from Reddy-Garlapati and Sodeifian models (Eqs. 24, 25), respectively.

**Figure 9:**
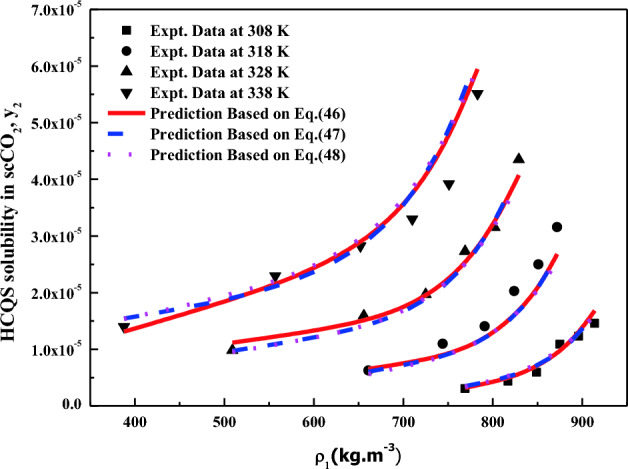
HCQS solubility in scCO_2_ versus $\rho_{1}$. Symbols and lines are experimental and calculated from new solvate complex models (Eqs. 46, 47, 48), respectively.

All the regression results are summarized in Table 3 and presented in Figs. 3, 4, 5, 6, 7, 8 and 9. Solid–liquid model requires enthalpy of fusion, molar volume and melting point of the solute. Enthalpy of fusion is estimated with the help Jain et al.^77^, and molar volume of the solute is estimated with the help Immirzi and Perini method^78^. The calculated molar enthalpy and molar volumes are 65,208 J mol^−1^ and 3.306 × 10^–4^ m^3^ mol^−1^, respectively. The melting temperature is obtained from the material safety data sheet as 240 °C. From Chrastil's model constant ($B_{1}$), total enthalpy is calculated ($B_{1}$ × R). From Bartle’s model constant ($B_{4}$), sublimation enthalpy is calculated ($\Delta_{sub} H = - B_{4} R$). From the magnitude difference between total and sublimation enthalpies, solvation enthalpy is calculated. Similarly, from reformulated Chrastil's model constant ($B_{2}$) and Bartle’s model constant ($B_{4}$), combination solvation enthalpy is calculated. All the calculated quantities are shown in Table 4. The regression ability of commonly used three parameter models are found to be inferior when compared to Sodeifian et al., and Reddy–Garlapati models. This may be due to a smaller number of parameters in the models; on the other hand, the correlating ability of the solvate complex models is quite good. However, among all empirical models, Reddy-Garlapati model is the best model. The newly proposed solvate complex models have more adjustable constants, and thus their predictions are also good. From the solvate complex model’s constants, it is interesting to note the behavior of association number. When association number is treated as a linear function of reduced density, the obtained average association number for the solubility data is $\kappa^{\prime\prime}_{ave}$ = 2.21, which is lesser than that of the conventional Chrastil's model $\kappa$ = 3.89. But, when association number is treated as quadratic function of reduced density, the obtained average association number for the solubility data is $\kappa^{\prime\prime}_{ave}$ = 3.78, and it is matching well with association number of Chrastil’s model. From those results and from literature arguments, we can infer that association number is quadratic function of $\rho_{r}$ so that is apparent than other forms and its correlations are reliable^71,79^. Thus, association number as quadratic function of $\rho_{r}$ is recommended for the data interpolation.

**Table 3:** Regression results of all models used in this research.

Model	Correlation parameters	AARD%	R²	R²_adj_
Solid–liquid equilibrium (SLE)	$\alpha$ = 4.9285 × 10^–3^_;_$\beta$ = − 1.156 × 10^–3^; $\lambda^{\prime}_{12}$ = 0.43761; $\lambda^{\prime}_{21}$ = 3.0828	17.27	0.927	0.912
Chrastil	$\kappa$ = 3.8918; $A_{1}$ = − 4.2291; $B_{1}$ = − 8391.6	24.27	0.829	0.804
Reformulated Chrastil	$\kappa^{\prime}$ = 3.8833; $A_{2}$ = − 18.92; $B_{2}$ = − 7457.9	24.32	0.828	0.803
Méndez-Santiago and Teja (MST)	$A_{3}$ = − 12,530; $B_{3}$ = 2.2133; $C_{3}$ = 25.435	26.50	0.779	0.769
Bartle	*A*_4_ = 26.917; $B_{4}$ = − 10,711; $C_{4}$ = 6.8092 × 10^–3^	27.04	0.787	0.777
Reddy-Garlapati	$A_{5}$ = 1.04535 × 10^–4^; $B_{5}$ = − 2.179 × 10^–5^; $C_{5}$ = 1.897 × 10^–5^; $D_{5}$ = − 1.4246 × 10^–4^ $E_{5}$ = 1.7054 × 10^–5^ $F_{5}$ = − 1.684 × 10^–5^	10.04	0.974	0.973
Sodeifian	$A_{6}$ = 8.6490; $B_{6}$ = − 1.0492 × 10^–3^; $C_{6}$ = 3.0286 × 10^–1^; $D_{6}$ = 7.1397 × 10^–4^ $E_{6}$ = 1.9898 × 10^–2^ $F_{6}$ = − 4.9173 × 10^2^	13.10	0.944	0.941
Solvate complex-Eq. (46)	$\kappa^{\prime\prime} = a_{1} + a_{2} \rho_{r} + a_{3} \rho_{r}^{2}$ where a1=0.58597;a2=2.6675 a3=− 0.40881 andL=− 2207.3 M= − 0.018958;N=− 4.6158 $\kappa^{\prime\prime}_{ave}$ = 3.78	10.08	0.96	0.958
Solvate complex-Eq. (47)	$\kappa^{\prime\prime} = a_{1} + a_{2} \rho_{r}$ where a1=− 0.28155;a2=1.5579 and L= − 6206.5;M= − 0.015134;N=13.107 $\kappa^{\prime\prime}_{ave}$ = 2.21	12.00	0.955	0.951
Solvate complex- Eq. (48)	$\kappa$ = 3.089; L = − 4162;M = − 8.4529 × 10^–4^; N = − 8.4428	12.30	0.949	0.941
Rajasekhar and Madras-Eq. (49)	$\kappa^{\prime\prime\prime}$ = 3.089; $L^{\prime}$ = − 4162; $M^{\prime}$ = − 8.4529 × 10^–4^; $N^{\prime}$ = − 8.4428	12.30	0.949	0.9411

**Table 4:** Enthalpies of sublimation and solvation of HCQS.

Model	Name of property		
	Total Enthalpy, ΔH_total_ (kJ/mol)	Enthalpy of sublimation ΔH_sub_(kJ/mol)	Enthalpy of solvation (kJ/mol)
Chrastil's model	69.80^a^		− 19.30^d^
Modified Chrastil's model	62.00^b^		− 27.10^e^
Bartle et al., model		89.1^c^ (approximate value)	

^d^Obtained as a result of difference between ΔH_sub_^c^ and ΔH_total_^a^.

^e^Obtained as a result of difference between ΔH_sub_^c^ and ΔH_total_^b^.

Further, a comparative analysis is done to determine the best model for HCQS-scCO_2_ system. Since, a varying number of parameters are involved in the equations considered in the work, Akaike Information Criterion (AIC) and corrected AIC (AIC_c_)^80–83^ are used to identify the best model. AIC alone is used when data points are more than forty; on the other hand, AIC_c_ is used when data points are less than forty. In the present there are only twenty-four solubility data points in our hands; hence, AIC_c_ is used for identifying the best model. AIC_c_ is defined as Eq. (52)

52 $$AIC_{c} = AIC + \frac{{2Q\left( {Q + 1} \right)}}{N - Q - 1}$$

where N is solubility data points, *Q* is number of model constants, and SSE is error sum of squares. From the least AIC_c_ value, the best model is identified. The lower the AIC_c_ value the greater the accuracy of the model, and it is independent of the number of parameter. All the AIC_c_ values are reported in Table 5. From results, Reddy-Garlapati and the new solvate complex models are observed to be the better models.

**Table 5:** Summary of SSE, RMSE, AIC and AIC_c_ of all solubility models.

Model	SSE (•10^10^)	RMSE (•10^6^)	*N*	*Q*	AIC	AIC_c_
Standard solubility models						
Chrastil's model	9.284	6.219	3	24	− 569.4	− 568.2
Modified Chrastil's model	9.349	6.241	3	24	− 569.2	− 568.0
Mendez-Teja model	6.935	5.375	3	24	− 500.1	− 498.4
Bartle et al., model	11.923	7.048	5	24	− 563.4	− 562.2
Reddy-Garlapati model	1.381	2.399	6	24	− 609.1	− 604.1
Sodeifian et al., model	3.956	4.060	6	24	− 583.9	− 579.7
Solvate complex models						
Equation (46)	1.99	14.124	6	24	− 600.0	− 595.4
Equation (47)	2.54	15.925	5	24	− 597.0	− 593.2
Equation (48)	2.55	15.961	4	24	− 598.0	− 596.3
Equation (49)	2.55	15.961	4	24	− 598.0	− 596.3

## Conclusion

Solubilities of solid HCQS in scCO_2_ solvent were measured at various conditions ranging from P = 12 to 27 MPa and T = 308 to 338 K. The measured data’s range is from 0.0304 × 10^–4^ to 0.5515 × 10^−4^ in terms of mole fraction. Three forms of solvate complex models explored in this study are reasonable in estimating solubility and among the three the best model is observed to have AICc and AARD values − 595.4 and 10.08% respectively. Among empirical models Reddy-Garlapati model is observed to fit the data quite well and with the corresponding AICc and AARD values − 604.1 and 10.04%, respectively.

## Data Availability

On request the data may be obtained from the corresponding author.

## List of symbols

*A*_*1*_, *B*_*1*_: Constants of Eqs. (19) and (20)
*A*_*2*_, *B*_*2*_: Constants of Eq. (21)
*A*_*3*_, *B*_*3*__,_*C*_*3*_: Constants of Eq. (22)
*A*_*4*_, *B*_*4*__,_*C*_*4*_: Constants of Eq. (23)
*A*_*5*_, *B*_*5*__,_*C*_*5*_, *D*_*5*_, *E*_*5*_, *F*_*5*_: Constants of Eq. (24)
*A*_*6*_, *B*_*6*__,_*C*_*6*_, *D*_*6*_, *E*_*6*_, *F*_*6*_: Constants of Eq. (25)
AARD%: Average absolute relative deviation percentage
AIC: Akaike Information Criterion
AIC_c_: Corrected *AIC*
C_s_: Drug in sample in vial (g/L)
E1 to E14: Symbols used in experimental setup
H_sol_: Solvation enthalpy (kJ/mol)
H_sub_: Sublimation enthalpy (kJ/mol)
H_total_: Total enthalpy (kJ/mol)
${\text M}_{\text {CO}_2}$, M_S_: Molar mass of CO_2_ and drug (g/mol)
${n}_{CO_2}$: Moles of carbon dioxide
${n}_{\mathrm{drug}}$: Moles of drug
*N*: Number of experimental data points
NIST: National Institute of Standards and Technology
OF: Objective function
*Q*: Number of parameters of a model
P: System pressure
P_c_, P_r_: Critical pressure, Reduced pressure
P_s_: Sublimation pressure (Pa)
*R*: Universal constant of ideal gas, 8.314 J/mol K
RMSD: Root mean square deviation
S: Solubility (g/L)
SSE: Sum of squares error
scCO_2_: Supercritical carbon dioxide
T: System temperature (K)
T_c_: Critical temperature (K)
$v_{1} ,v_{2} ,v_{s}$: Molar volume of solvent, solute and solute (m^3^/mol)
*V*_*1*_* ,**Vs*: Sampling loop and collection vial volumes (μL)
$y_{2}$: Drug solute solubility in mole fraction

## Greek symbols

$\alpha ,\alpha^{\prime}$: Constants in Eqs. (16) and (38), respectively
$\beta ,\beta^{\prime}$: Constants in Eqs. (16) and (38), respectively
$\rho ,\rho_{r}$: Density, reduced density
$$\kappa,\kappa^{\prime},\kappa^{\prime\prime},\kappa^{\prime\prime\prime}$$: Association numbers
$\gamma_{2}^{\infty }$: Infinitesimally dilute state activity coefficient of solute
$\lambda^{\prime}_{12} ,\lambda^{\prime}_{21}$: Energies of interaction
